# Evaluation protocol for amusia - portuguese sample

**DOI:** 10.5935/1808-8694.20120039

**Published:** 2015-10-20

**Authors:** Maria Conceição Peixoto, Jorge Martins, Pedro Teixeira, Marisa Alves, José Bastos, Carlos Ribeiro

**Affiliations:** a MD. ENT resident - University Hospital of Coimbra); b MSC; Audiologist - University Hospital of Coimbra); c Professor of Music - Colégio do Rosário - Porto); d MSc. Speech Therapist - University Hospital of Coimbra); e MD. ENT assistant physician - University Hospital of Coimbra); f Head of the ENT at the University Hospital of Coimbra)

**Keywords:** hearing disorders, diagnosis, disability evaluation, auditory diseases, central

## Abstract

Amusia is a disorder that affects the processing of music. Part of this processing happens in the primary auditory cortex. The study of this condition allows us to evaluate the central auditory pathways.

**Objective:**

To explore the diagnostic evaluation tests of amusia.

**Method:**

The authors propose an evaluation protocol for patients with suspected amusia (after brain injury or complaints of poor musical perception), in parallel with the assessment of central auditory processing, already implemented in the department. The Montreal Evaluation of Battery of amusia was the basis for the selection of the tests. From this comprehensive battery of tests we selected some of the musical examples to evaluate different musical aspects, including memory and perception of music, ability concerning musical recognition and discrimination. In terms of memory there is a test for assessing delayed memory, adapted to the Portuguese culture. Prospective study.

**Results and Conclusions:**

Although still experimental, with the possibility of adjustments in the assessment, we believe that this assessment, combined with the study of central auditory processing, will allow us to understand some central lesions, congenital or acquired hearing perception limitations.

## INTRODUCTION

Studies concerning musical changes started in the field of neuropsychology. In 1865, immediately after Broca described the first case of language change as a result of a lesion on the frontal area of the left hemisphere, Bouillaund described the first series of cases in which numerous musical skills were lost in consequence to a brain injury. This is commonly referred to as amusia^1^.

Amusia is a dysfunction which impairs musical processing, although it may also involve memory and musical knowledge.

It may be congenital or acquired. The acquired forms encompass all of those brain injuries which lead to a loss in the person's capacity to produce musical sounds, but it spares speech. The congenital form corresponds to a hereditary alteration characterized by impaired musical perception skills without any other associated cognitive deficit, hearing loss, brain lesions or lack of exposure. Although many bad singers consider themselves as having “bad ears”, it is estimated that about 4% of the population are affected by a neurogenetic alteration called congenital amusia^2^.

Patients with this disorder have an inability to recognize a given musical tone or misperceive the notes in a known song and they usually are people who do not take any interest in music and say that they “do not perceive anything associated with music”^3^. They are unable to perceive or hum familiar songs, despite having normal audiometry results and having normal or above average intellectual and memory capacity. They also frequently fail in recognizing subtle pitch differences lower than a semi-tone and are usually bad singers, despite having normal speech capacity, which is the contrary of their perception capacities^4^.

Current studies have shown dissociation in rhythm, melody and emotional processing of music, and amusia may represent insufficiency in any combination of these sets of skills^5^.

Clinical presentation is variable, thanks to the dependance on different variables.

The symptoms associated with amusia are usually classified into receptive, clinical or mixed.

The symptoms associated with reception amusia, often times referred to as “musical deafness” include the incapacity to recognize known melodies, lack of skills to read musical notation, incapacity to detect errors or incorrect musical notes in a melody. The clinical or expressive symptoms include the loss of a capacity to sing, write musical notation and/or play an instrument. The mixed disorder would be a combination of the expressive and the receptive involvements^6^, ^7^.

The diagnosis of amusia may be achieved by using the *Montreal Battery of Evaluation of Amusia* (MBEA), which involves a number of tests assessing known musical traits as contributors for musical memory and perception. This battery involves six tests which assess the capacity to discriminate pitch, musical scales, pitch intervals (tuning), rhythm, periodical stressing and memory^8^.

Although of undeniable usefulness in terms of investigation, its application in clinical practice is very difficult because of the slowness in assessment and the degree of difficulty in some cases, considering the Portuguese population.

Beyond this aspect, knowing that in the processing of musical information we use the primary auditory cortex, the secondary auditory cortex and the limbic system, it seems interesting to assess these structures in other ways which are not only the pure tone or vocal sounds^6^.

The goal of this study was to explore the amusia diagnosis tests and assess their value in daily clinical practice.

## METHOD

Based on the tests created by the MBEA, we tried to create a protocol, adapted and adjusted to the daily clinical practice in order to assess the changes in music processing.

The MBEA is based on six tests. The tests try to assess, in total, three musical components: the melody organization, the temporal organization and the recognition or memory, enabling the functional assessment of each one of the musical components.

All the six tests use the same group of 30 musical phrases, which were made up according to the guidelines set by the *Western Tonal System*. The selections last from 3.8 to 6.4 seconds, except in the periodic stressing test – the *Metric Test*, in which the stimulus lasts twice as much. Along each one of the tests we carry out certain manipulations in these musical phrases, trying to analyze the change being studied.

Pitch variations include the tone assessment tests – *scale alternate*, *contour alternate* and *interval alternate*.

The pitch test assesses one's capacity of recognizing a change in pitch, so as to have it outside of the scale, as we maintain the pitch of the original melody.

The test of change to the melodical line is created by the change of a critical pitch, so as to change the direction of the general pitch of the melody.

The interval changing test is based on the change in the distance between two adjacent semitones.

The temporal organization involves the – *rhythm alternate* test and the *metric test*.

The rhythm change test was created by the change in the duration of a given figure within the musical phrase.

The periodic stress assessment test aims at assessing the capacity to recognize a given pace, in this particular study, binary or ternary. For the periodic stress test we used sequences of two musical phrases.

Thus, we try to create different comparison and distinction patterns within the assessment criteria proposed by the different tests.

Vis-à -vis the assessment of memory, we considered two parts: one to assess the recent memory, such as the one proposed by the MBEA, with the recognition of music presented throughout the remaining test, and the other to assess the past memory, using music that is known to the public in general, such as “Happy Birthday to You”, made and recorded by one of the authors, following the same rules of composition used in the MBEA.

Given the extension of the tests proposed by the MBEA, we selected for this protocol the 10 first musical phrases created by the MBEA in each one of the tests. We kept the same answer model used in the original study battery.

## RESULTS

We created a protocol based on the MBEA, trying to adjust it to clinical practice, but without changing the standards used to validate it.

**Figure 1:**
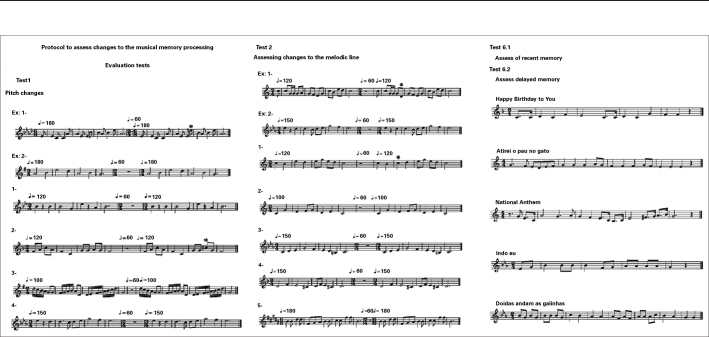
Example of the protocol used in the assessment of music processing - part of the test 1, 2 and 6.

**Figure 2:**
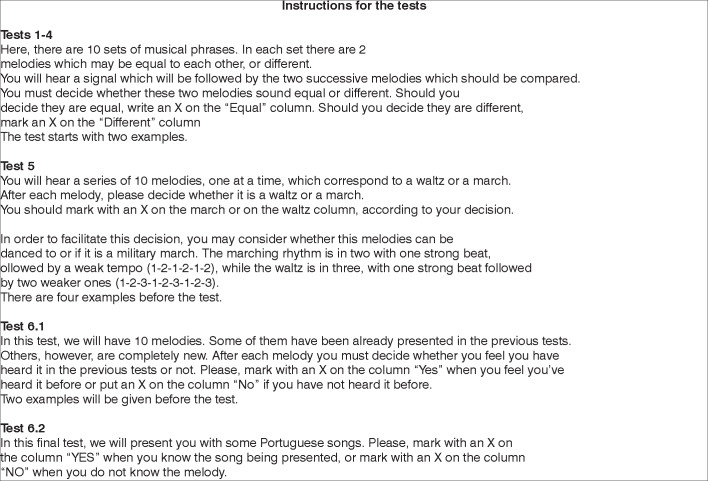
Instructions given to perform the tests.

**Figure 3:**
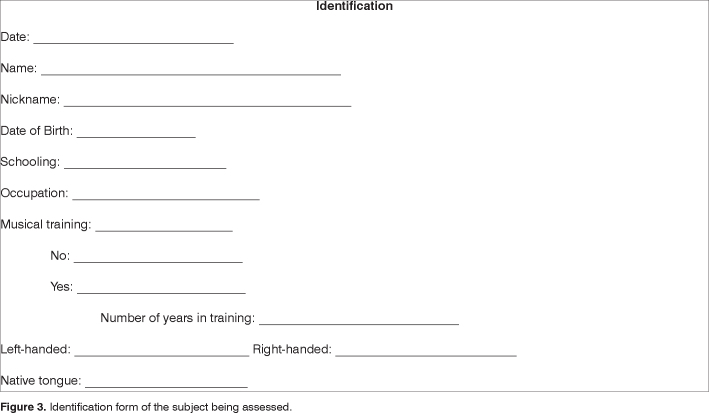
Identification form of the subject being assessed.

**Figure 4:**
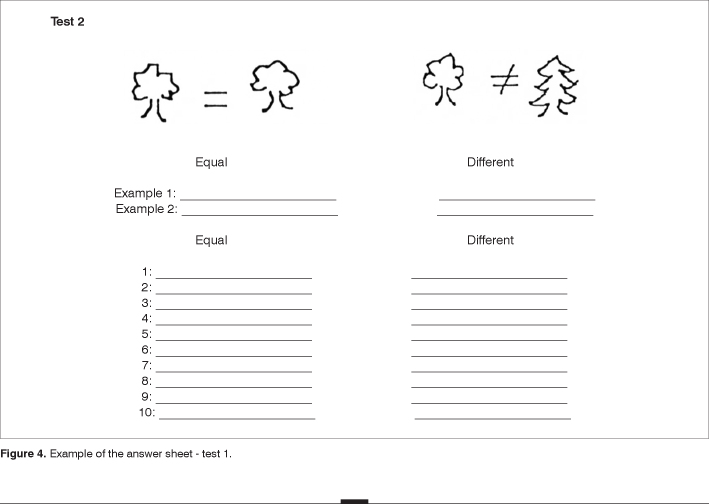
Example of the answer sheet - test 1.

The adapted protocol used is presented on Figures 1, 2, 3, and 4.

## DISCUSSION

Humans seem to be musical beings by birth. Even before 1 year of age, children have evolved musical perception, similar to that of adults. In particular, children have increased capacities to process changes in pitch and in regular rhythms. This initial preference for coding along musical scales and to assign a regular pulse to events are essential for the hierarchical organization of music. This type of organization will ease the musical processing by creating expectations and feelings of surprise and satisfaction^9^.

Nonetheless, some individuals seem to not have this predisposition towards music. Four to five percent of the population have a congenital lack of musical skills, and such lack of musical capacity can not be explained by any deficit of intelligence or lack of environmental exposure^9^, ^10^.

By the same token, multiple alterations to the musical capacities happen after a given brain damage. The opposite is also true. Early brain injuries or even broad brain lesions may, sometimes, spare musical skills in patients suffering from important cognitive losses. Another important aspect is the fact that a brain lesion may very selectively affect musical capacities, while the rest of the cognitive system, including language, remains intact. And, moreover, not all musical skills are equally affected. It is known that a damage to the left hemisphere may spare the capacity for melodical representation in terms of contour, but interfering with alterations in rhythm - changing the interval, while a damage to the right hemisphere impacts on both functions. Another interesting example is dementia. It is clearly known that patients with dementia maintain a level of musical knowledge and pleasure with musical activities which may not be compared to other activities, even in more advanced stages of the disease. And finally, about 10% of the general population have learning limitations in particular domains, such as in reading and language, and one example is dyslexia. It is very interesting to know whether these limitations are extended to the musical domain. And it is already known that those with congenital amusia have a compromised performance in the identification, discrimination and imitation of phrases with different intonations, especially in the final words. This suggests that amusia may harm the subtle processing of language^8^, ^11^, ^12^, ^13^, ^14^.

In order to document these changes, it is necessary to use behavioral tests.

So far, the best assessment tool available is the *Montreal Battery of Evaluation of Amusia* (MBEA). Throughout the last decade, this battery has been developed and validated in populations complaining of auditory processing, as well as in populations with brain injuries of different etiologies.

Its creation is based on a model of musical perception and memory. In such model, one tries to analyze the melody (defined by sequential variations in pitch), the temporal dimension (defined by sequential variations in duration) and, finally, trying to map, within prior representations, whether there is any corresponding, in an attempt to assess the musical memory.

One fundamental aspect is to consider that the received auditory input will activate the musical component or the language processing system associated with the lexical representation. The musical component, which we discuss in this paper, will be later analyzed on its melodical component, aiming at answering the question “what” and about its temporal component, aiming at answering the question “when?”^15^.

Different cortical areas are involved in the analysis of temporal and melodic components.

Studies suggest that the pitch analysis is primarily controlled by the right temporal region. The right-side secondary auditory cortex processes pitch changes and the fine manipulation of tone. This region distinguishes multiple pitches which characterize the melody in terms of contour (pitch direction) and interval (relationship of frequencies between successive notes). The right-side superior temporal gyrus assesses the contour information while both right and left temporal regions recruit and assess temporal information. Moreover, the primary auditory cortex is also involved in processing pitch information.

In regards of the temporal components, these are analyzed in two ways: by means of segments of continuous sequences of music, in temporal events based on duration, and by means of grouping temporal events which enable the understanding of the rhythm underlying the music. Studies on rhythmic discrimination have shown that the right-side temporal auditory cortex is responsible for temporal segmentation and the left-side temporal auditory cortex is responsible for temporal grouping. Other studies suggest the participation of areas from the motor cortex in this analysis. Thus, the lack of involvement and the connection between the bilateral temporal cortexes and the motor centers may contribute both to the congenital amusia as well as to its acquired form^13^, ^16^, ^17^.

And, finally, we still have to assess memory. Memory is necessary to process and integrate both melodical and rhythmical aspects of music. Studies suggest that there is an interconnection between the right-side temporal gyrus and the frontal cortical areas for the working memory in musical appreciation. This connection between the temporal and frontal regions of the brain is extremely important, since these regions play critical roles in music processing. Changes in the temporal areas are probably due to deficits in the perception of pitch and other musical characteristics, while changes in the frontal area are potentially associated to failures in aspects of cognitive processing, such as memory. Memory is also a preoccupation with the internal representation and recognition of songs, which help identify known songs and check the skill of being able to sing these songs. The activation of the superior temporal region, the left inferior temporal region and the frontal region is responsible for the recognition of known songs^9^, ^18^, ^19^.

The assessment with MBEA is carried out in one single session, lasting for approximately 1.5 hour, without resting intervals. The order of presentation is fixed and starts with the scale or tonality tests, contour or alteration on the melodical line, intervals, followed by rhythm, metrics or pace tests, and memory tests. An individual is considered amusic when placed at two standard-deviations below the average obtained by music-competent controls^8^.

Although these are validated and standardized tests, without doubts fulfilling the role they were designed to fulfill, their use in clinical practice is very difficult. For such end, we looked for tests which are easier to understand, and with high sensitivity and specificity, but tests which are doable within the limits imposed in terms of duration of execution and time needed for interpretation.

Concerning the disorders alluded to, there are no questions concerning the applicability of the MBEA in those patients with neurological impairments, as well as in patients with congenital amusia. However, we still need to define the role of such assessment in singing, in those patients who are unable to sing or have bad performance in singing, although with good capacity of musical analysis, as well as in the specific fields of learning.

The identification of individuals with amusia takes on a particular interest, considering that even in individuals with congenital amusia or in their descendants, a greater environmental exposure may mitigate musical disabilities. We may then identify and propose a framework of training which may reduce the difficulties presented^8^.

Having said that, we consider that the application of these tests, even when adapted, may help in the assessment of these aspects. In these regards, we are also developing assessment questionnaires in our department to evaluate musical perception and recognition.

## CONCLUSIONS

The study of amusia is based on the assessment of changes to music perception. This analysis enables us to study different brain lesions, as well as to assess changes in singing. It also enables us to analyze, in parallel, the central auditory pathways, which are common to the perception of music in many of its aspects.
